# Bidirectional Control of Orienting Behavior by the Substantia Nigra Pars Reticulata: Distinct Significance of Head and Whisker Movements

**DOI:** 10.1523/ENEURO.0165-21.2021

**Published:** 2021-10-20

**Authors:** Sebastian Hormigo, Ji Zhou, Manuel A. Castro-Alamancos

**Affiliations:** Department of Neuroscience, University of Connecticut School of Medicine, Farmington, CT 06001

**Keywords:** basal ganglia, orienting behavior, substantia nigra, vibrissa, whisker

## Abstract

Detection of an unexpected, novel, or salient stimulus typically leads to an orienting response by which animals move the head, in concert with the sensors (e.g., eyes, pinna, whiskers), to evaluate the stimulus. The basal ganglia are known to control orienting movements through tonically active GABAergic neurons in the substantia nigra pars reticulata (SNr) that project to the superior colliculus. Using optogenetics, we explored the ability of GABAergic SNr neurons on one side of the brain to generate orienting movements. In a strain of mice that express channelrhodopsin (ChR2) in both SNr GABAergic neurons and afferent fibers, we found that continuous blue light produced a robust sustained excitation of SNr neurons which generated ipsiversive orienting. Conversely, in the same animal, trains of blue light excited afferent fibers more effectively than continuous blue light, producing a robust sustained inhibition of SNr neurons which generated contraversive orienting. When ChR2 expression was restricted to either GABAergic SNr neurons or GABAergic afferent fibers from the striatum, blue light patterns in SNr produced only ipsiversive or contraversive orienting movements, respectively. Interestingly, whisker positioning and the reaction to an air-puff on the whiskers were incongruent between SNr-evoked ipsiversive and contraversive head movements, indicating that orienting driven by exciting or inhibiting SNr neurons have different behavioral significance. In conclusion, unilateral SNr neuron excitation and inhibition produce orienting movements in opposite directions and, apparently, with distinct behavioral significance.

## Visual Abstract

**Figure:**
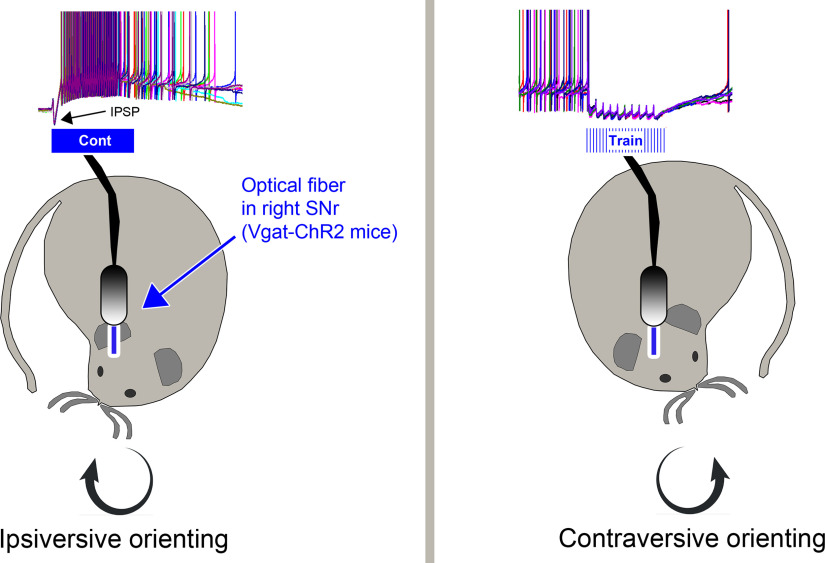

## Significance Statement

The substantia nigra pars reticulata (SNr) is the main output nucleus of the basal ganglia in the midbrain and is known to be involved in orienting behavior. We found a way to bidirectionally control the firing of SNr cells in same animal. This approach revealed that excitation or inhibition of these cells controlled the direction of orienting head movements in opposite directions and, apparently, with different behavioral significance.

## Introduction

In alert animals, an unexpected, novel or salient sensory stimulus leads to a well-known orienting response by which animals move their sensors in the direction of the stimulus to evaluate it (Sokolov, 1963). Orienting is important to identify stimuli that call for action; a decision to act (e.g., approach, escape, ignore) usually follows an orienting response. The superior colliculus has been intensely studied as an essential component of the neural circuitry controlling orienting. In fish and amphibians, the optic tectum (the equivalent of mammalian superior colliculus) is the principal structure responsible for spatial orienting (Marín et al., 1997; Suzuki et al., 2019). In mammals, many studies have investigated the contribution of the superior colliculus to orienting movements (Gandhi and Katnani, 2011), which is under inhibitory control from the basal ganglia through its output nucleus in the substantia nigra pars reticulata (SNr). In head-fixed primates, eye orienting movements (saccades) generated by the superior colliculus are regulated by the SNr (Hikosaka and Wurtz, 1983a,b; Sparks, 1986; Wurtz and Hikosaka, 1986; Hikosaka et al., 2000). In mice, SNr neurons map the position of head movements by modulating their firing as a function of movement direction (Barter et al., 2015; Yin, 2017). Moreover, modulation of SNr firing by direct and indirect striatonigral pathways controls the generation of movement in an open field, including the direction of reaching forelimb movements (Freeze et al., 2013; Yttri and Dudman, 2016). Thus, GABAergic SNr neurons may contribute to the generation of orienting movement direction as mice freely explore the environment. Early work revealed the effects of unilateral manipulations (e.g., drug infusions) in the ventral midbrain on rat rotational behavior (Tarsy et al., 1975; Olianas et al., 1978; Di Chiara et al., 1979; Kilpatrick and Starr, 1981; Kilpatrick et al., 1982; Leigh et al., 1983), unilateral inhibition of the substantia nigra (e.g., muscimol) causes contraversive rotation while excitation (e.g., disinhibition with picrotoxin) causes ipsiversive rotation.In contrast to drug infusions or other traditional methods, optogenetics (Boyden et al., 2005) allows time-selective and neuron specific excitation or inhibition of GABAergic SNr neurons or their afferents. Thus, using optogenetics in mice of either sex, we explored the effect of manipulating GABAergic SNr neuron firing on exploratory head and whisker movements in an open field.

## Materials and Methods

### Experimental design and statistical analysis

All procedures were reviewed and approved by the institutional animal care and use committee and conducted in adult (more than eight weeks) male and female mice. Experiments involved a repeated measures design in which the mice or cells serve as their own controls (comparisons within groups), but we also compared experimental mice to No Opsin controls and different groups of experimental animals to each other as noted (comparisons between groups). For comparisons within groups, we tested for a main effect using a mixed design ANOVA followed by comparisons with Tukey’s test. In this mixed design ANOVA, the stimulus condition was the main effect (with as many levels as conditions tested; e.g., blue light patterns). The sessions were treated as random. In a balanced design, this is equivalent to a linear mixed-effects model in which Stimulus is a fixed-effect and Sessions are a random-effect nested within the subjects [data ∼stimulus + (1|subjects/sessions), as per lme4 syntax in R]. For comparisons between different groups, we used the same approach but included the group as an additional fixed-effect [data ∼group × stimulus + (1|subjects/sessions)]. Using the standard errors derived from the model, Tukey’s tests were conducted for the effect of the Stimulus (within group comparisons) or for the group-stimulus interaction (between group comparisons). We report the Tukey’s values for the relevant multiple comparisons. In addition, we performed the same comparisons (both within groups and between groups) using a bootstrap approach by randomly sampling with replacement (1000–10,000 times) from all the values (regardless of the conditions or groups) and determining the probability that the difference (or a larger difference) between the conditions or groups occurs by chance (these *p* values are reported inside brackets, []). A power analysis conducted with OriginLab Pro using the measured means difference variability revealed that three animals in which we conducted approximately five identical daily sessions per animal (15 sessions in three mice) was sufficient to detect a significant change (∼10° change in head bias or an ∼20% change in avoidance rate) with a power of 0.99 (*p* = 0.05). This was used as the bare minimum number of animals and sessions per group. Thus, the *N*s for the behavioral experiments correspond to the daily sessions in which all the experimental stimuli (conditions tested) were applied to allow repeated comparisons within the session (≥30 repetitions of each stimulus per session). For the whole-cell recordings in slices, the *N*s correspond to cells in different slices and mice in which the protocols were compared using a repeated measures ANOVA.

To enable rigorous approaches, we maintain a local server with a central relational database accessed through a wiki that logs all details and metadata related to the experiments, including all information about animals and details about surgical procedures, behavioral sessions, electrophysiological recordings, histology, and scripts used for analyses. Moreover, during daily behavioral sessions, computers run experiments automatically using preset parameters logged for reference during analysis. Analyses are performed using scripts that automate all aspects of data analysis from access to metadata and data files to population statistics and graph generation (scripts and metadata will be accessible through our website or by request).

### Strains and adeno-associated viruses (AAVs)

The following AAVs (injected undiluted) and mouse strains were used in the present study to manipulate the activity of neurons that express channelrhodopsin (ChR2) or Arch with optogenetics. To generate Vgat-ChR2 mice that express ChR2 in GABAergic cells, including both SNr cells and afferents, we crossed Vgat-cre (Jax 028862; B6J.129S6(FVB)-Slc32a1^tm2(cre)Lowl^/MwarJ) and Ai32 (Jax 024109; B6.Cg-Gt(ROSA)26Sor^tm32(CAG-COP4*H134R/EYFP)Hze^/J). To selectively excite GABAergic SNr cells, we injected Vgat-cre mice with AAV5-EF1a-DIO-hChR2(H134R)-eYFP (UPenn Vector Core or Addgene, titers: 1.8 × 10^13^ GC/ml by quantitative PCR) in the SNr to express ChR2 in GABAergic SNr cells. To directly inhibit SNr GABAergic cells, we expressed Arch (using two different approaches) or IC++. In the Vgat-SNr-Arch group, we injected Vgat-cre mice with AAV5-EF1a-DIO-eArch3.0-EYFP (UNC Vector Core, titers: 3.4 × 10^12^ GC/ml by Dot Blot) in the SNr to selectively express eArch3.0 in SNr GABAergic cells. In the Vgat-Arch group, we crossed Vgat-cre and Ai40D (Jax 021188; B6.Cg-Gt(ROSA)26Sor^tm40.1(CAG-aop3/EGFP)Hze^/J) mice to express ArchT broadly in GABAergic cells. In the Vgat-SNr-IC++ group, we injected Vgat-cre mice with AAV5-EF1a-DIO-iC++-eYFP (UNC Vector Core, titers: 3.4 × 10^12^ GC/ml by Dot Blot) in the SNr to selectively express IC++ in SNr GABAergic cells. To excite GABAergic fibers in SNr and indirectly inhibit SNr neurons, we injected Vgat-cre mice with AAV5-EF1a-DIO-hChR2(H134R)-eYFP (UPenn Vector Core or Addgene, titers: 1.8 × 10^13^ GC/ml by quantitative PCR) in the striatum or nucleus accumbens (Acb) to express ChR2 in GABAergic striatum or Acb neurons. As a No Opsin control, we injected AAV8-hSyn-EGFP (Addgene, titers: 4.3 × 10^12^ GC/ml by quantitative PCR) in the SNr.

### Surgeries

Optogenetics experiments involved injecting 0.3-μl AAVs per site during isoflurane anesthesia (∼1%). Animals received carprofen after surgery. The stereotaxic coordinates for injection sites (in mm from bregma; lateral from the midline; ventral from the bregma-λ plane) are: SNr (3.3 posterior; 1.4–1.5; 4–4.1), dorsal striatum (StrD; 1.1 anterior; 1.8; 2.5), ventral striatum (StrV; 1.1 anterior; 1.8; 4), and Acb (1.2 anterior; 0.8; 4.3). The coordinate ranges reflect different animals that were grouped together because the slight coordinate differences produced similar effects.

In optogenetics experiments, a single or dual optical fiber (200 μm in diameter) was implanted unilaterally or bilaterally during isoflurane anesthesia at the above-mentioned coordinates and held in place with a combination of screws, cyanoacrylate, and dental cement. Optical fiber cannulas were implanted in the SNr at these coordinates (in mm): SNr (3.3 posterior; 1.5; 4.1).

### Optogenetics

The implanted optical fibers were connected to patch cables using sleeves. A black aluminum cap covered the head implant and completely blocked any light exiting at the ferrule’s junction. Furthermore, the experiments occurred in a brightly lit cage that made it difficult to detect any light escaping the implant. The other end of the patch cables was connected to a dual light swivel (Doric Lenses) that was coupled to a green laser (520 nm; 100 mW) to activate Arch or a blue laser (450 nm; 80 mW) to activate ChR2 or IC++. Unless otherwise noted, the behavioral experiments employed green light between 25–35 mW and blue light between 1 and 3 mW. Power is regularly measured by flashing the connecting patch cords onto a light sensor – with the sleeve on the ferrule.

### Orienting during open field exploration

Mice were placed in a circular open field (10’’ diameter) that was illuminated from the bottom to easily visualize whiskers and track movements (100 frames per sec (FPS)). We automatically tracked head movements with two color markers attached to the head connector; one located over the nose and the other between the ears. These coordinates were used to derive the head angle (midline). Whisker movements were tracked manually offline frame by frame by marking a well-defined whisker at the base (proximal) and at about ¾ of its length (distal). The angle of the whiskers versus the head angle were calculated using these coordinates. EMG of the whisker pad was employed to complement the whisker tracking. The mice had a pair of wires implanted under the whisker pad, as previously described for rats (Castro-Alamancos, 2006). The differential recording between each pair of wires was rectified and low pass filtered (100 Hz).

To stimulate the whiskers during active exploration of the arena, we used a long tube (29 gauge) connected to a solenoid valve that reached the cage floor from above. An observer manually moved the tube to place it in front of the left or right whiskers (∼1 cm away) as the animal moved about the cage. When the position was correct, the observer triggered a LabVIEW program controlling a NI USB-6363 to apply the optogenetic light and the air-puff (50 ms; 30 PSI) to deflect the whiskers. The random trials consisted of the air-puff alone, the blue light alone, or the air-puff and blue light delivered together.

### Active avoidance task in a shuttle box

Vgat-ChR2 mice were trained in a signaled active avoidance task, as previously described (Hormigo et al., 2016, 2019). During an active avoidance session, mice are placed in a standard shuttle box (16.1’’ × 6.5’’) that has two compartments separated by a partition with side walls forming a doorway that the animal has to traverse to shuttle between compartments. A trial consists of a 7-s avoidance interval followed by a 10-s escape interval. During the avoidance interval, an auditory conditioned stimulus (ACS, 8 kHz, ∼85 dB) is presented for the duration of the interval or until the animal produces a conditioned response (avoidance response) by moving to the adjacent compartment, whichever occurs first. If the animal avoids by moving to the next compartment, the CS ends, the escape interval is not presented, and the trial terminates. However, if the animal does not avoid, the escape interval ensues by presenting white noise and a mild scrambled electric foot-shock (0.3 mA) delivered through the grid floor of the occupied half of the shuttle box. This unconditioned stimulus (US) readily drives the animal to move to the adjacent compartment (escape response), at which point the US terminates, and the escape interval and the trial ends. Thus, an avoidance response will eliminate the imminent presentation of a harmful stimulus. An escape response is driven by presentation of the harmful stimulus to eliminate the harm it causes. In principle, successful avoidance is highly adaptive because it warrants the absence of harm. Each trial is followed by an intertrial interval (duration is randomly distributed; 25- to 45-s range), during which the animals await the next trial and are free to cross between compartments; there was no consequence for intertrial crossings.

### Behavioral measures and video tracking in the shuttle box

There are three main variables representing task performance in the shuttle box. The percentage of active avoidance responses (% avoids) represents the trials in which the animal actively avoided the US in response to the CS. The response latency (latency) represents the time (seconds) at which the animal enters the safe compartment after the CS onset; avoidance latency is the response latency only for successful active avoidance trials (excluding escape trials). The number of crossings during the intertrial interval (intertrial crossings) represents random shuttling because of locomotor activity.

Animals are also video tracked (30 FPS) during active avoidance sessions. The tracking followed color markers located on the head connector above the nose and between the ears. Several movement (tracking) measures were derived during active avoidance. Distance was the number of pixels crossed by the animal in its trajectory during the avoidance and escape intervals of a trial (trial distance) or during the intertrial interval (intertrial distance). Trial speed was the trial distance divided by the response latency. Intertrial speed was the intertrial distance divided by the intertrial interval duration. Trial velocity was the displacement divided by the response latency (displacement was the number of pixels in a straight line between the position of the animal at trial start and the position of the animal at trial end when the animal avoided or escaped). Pixel measures were converted to metric units using calibrations. Trial speed and intertrial speed represent the overall movement of the animal in any direction during those periods, while trial velocity represents movement in the correct direction to avoid.

### *In vitro* slice recordings

For slice preparation, adult mice (more than six weeks) were deeply anesthetized with an overdose of ketamine. Upon losing all responsiveness to a strong tail pinch, the animal was decapitated and the brain was rapidly extracted. Slices (400 μm thick) were cut in the coronal or sagittal plane using a vibratome at the level of SNr. Slices were transferred to an interface chamber where they were bathed constantly (1–1.5 ml/min) with artificial CSF (ACSF) at 32.5°C. The ACSF contained the following: 126 mm NaCl, 3 mm KCl, 1.25 mm NaH_2_Po_4_, 26 mm NaHCO_3_, 10 mm dextrose, 2 mm MgSO_4_ 7H_2_O, and 2 mm CaCl_2_ 2H_2_O. Blind whole-cell recordings were obtained from the SNr using patch electrodes of 4- to 10-MΩ impedance. The electrodes were filled with internal solution (290 mOsm) containing the following: 135 mm K-gluconate, 4 KCl, 2 mm NaCl, 0.2 mm EGTA, 10 mm Tris-phosphocreatine, 0.3 mm trisGTP, 10 mm HEPES, and 4 mm MgATP Each slice was imaged using a compound fluorescent microscope to reveal the parts of the brain that contained labeled cells/fibers. This also allowed verification of the correct AAV injection placement. At the end of each recording, the slice was imaged again to record the location of the whole cell recording electrodes. The internal solution contained neurobiotin (0.2%) to label the recorded cells, which were reconstructed if successfully labeled.

In slice experiments, an optical fiber (200 μm) coupled to an LED with a patch cable was used to apply pulses of blue light (470 nm; 0.1–3 mW) at the whole-cell recording site. For population data, we report the effects of the estimated power delivered on average in vivo (∼1.7 mW). Per trial, trains of 1-ms pulses (5–100 Hz) and continuous pulses of light were delivered (at least 4 s apart) in a sequence so that comparison between different light stimuli is done under equal conditions of the recorded cell. The effect of light on cell firing was tested both on spontaneous firing (if present) at resting membrane potential (Vm) and on firing induced by an 800-ms positive current pulse triggered 200 ms before light onset. To confirm the GABAergic nature of evoked IPSPs, a GABA_A_ receptor antagonist was added to the ACSF (bicuculline methiodide; 10 μm).

### Histology

Mice that did not undergo slice recordings were deeply anesthetized with an overdose of ketamine. Upon losing all responsiveness to a strong tail pinch, the animal was decapitated and the brain was rapidly extracted and placed in fixative. The brain was sectioned (100-μm sections) in the coronal or sagittal planes. Sections were mounted on slides, cover-slipped with DAPI mounting media, and photographed using a fluorescent microscope.

## Results

### Bidirectional control of SNr neuron firing in Vgat-ChR2 mice

**Figure 1.**
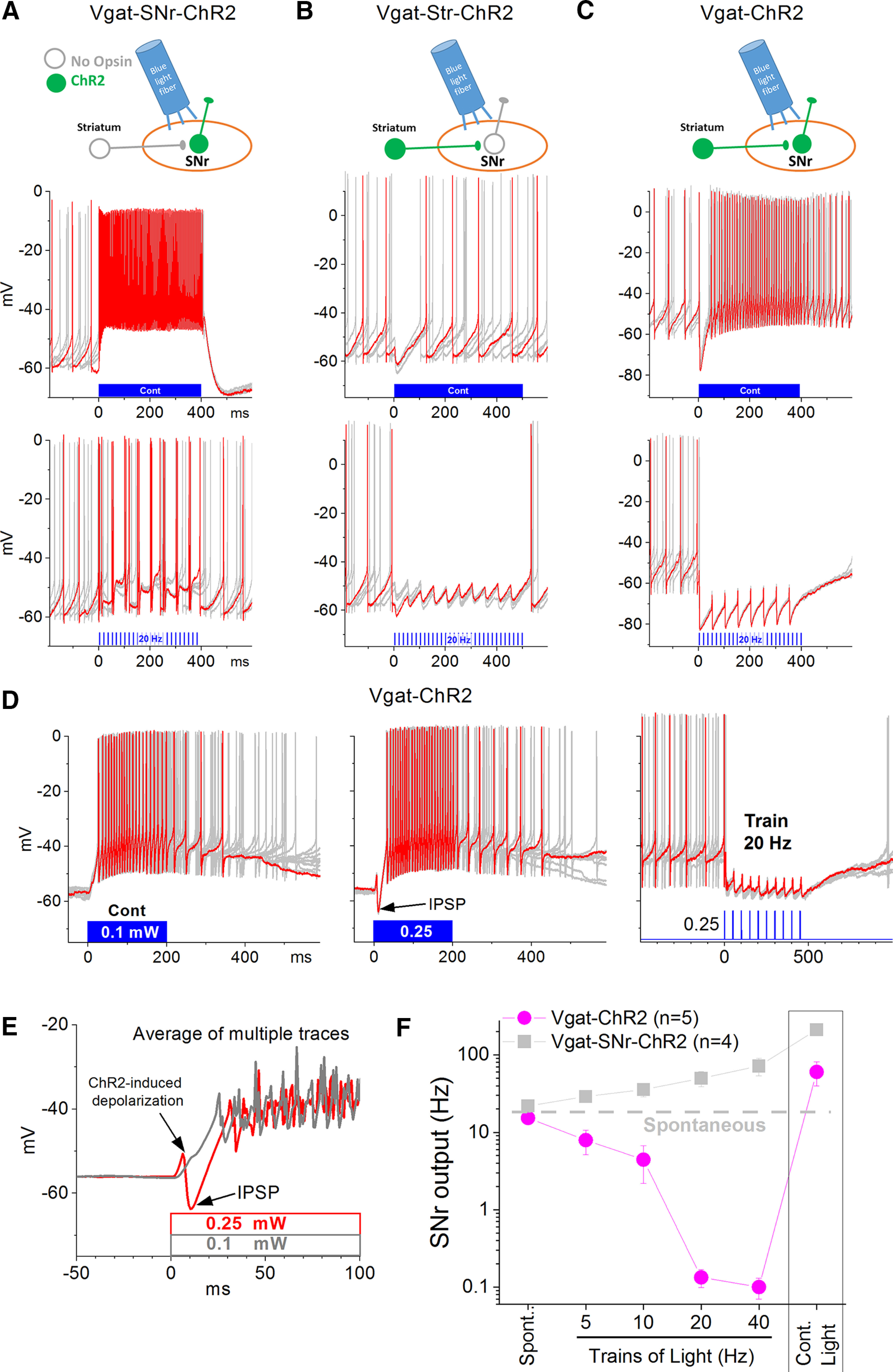
Effect of optogenetic blue light patterns on SNr firing in slices. ***A–C***, Whole-cell recordings from SNr cells in slices of Vgat-SNr-ChR2 (***A***), Vgat-Str-ChR2 (***B***), and Vgat-ChR2 (***C***) mice. Each panel overlays five trials showing the effect of continuous blue light (upper) and of a blue light train (lower, 20 Hz; 1-ms pulses) applied in SNr (each panel highlights one trial in red). The schematic indicates in green the expression of ChR2 in GABAergic cells of either the SNr (***A***), the striatum (***B***), or both (***C***). Note that in Vgat-ChR2 mice, the continuous pulse excited the SNr cell, while the train inhibits the SNr cell, which is a combination of the effects evoked in the other mice. ***D***, Effect of continuous blue light at different powers (0.1 vs 0.25 mW; left and middle panels), which reveals an IPSP at the onset of the pulse when the power is increased. Application of a train (20 Hz; 1-ms pulses) at the same 0.25-mW power drives a sustained IPSP that abolishes SNr firing. Each panel highlights one trial in red. ***E***, Overlay of averaged traces at the onset of continuous blue light at the two different powers reveal the IPSP evoked by the light at the higher power. ***F***, Population data from whole-cell recordings showing the effect of different patterns of blue light on the firing of SNr cells in slices from Vgat-ChR2 and Vgat-SNr-ChR2 mice. In Vgat-SNr-ChR2 mice, which express ChR2 selectively in SNr GABAergic cells, blue light produces an increase in SNr firing as a function of train frequency that becomes maximal during continuous blue light. In contrast, in Vgat-ChR2 mice that also express ChR2 in GABAergic afferents to SNr, blue light applied as trains produces a suppression of SNr firing while continuous blue light excites SNr firing.

In ChR2-expressing membranes, application of continuous blue light produces a fast cation current that desensitizes and stabilizes at a sustained steady-state (Nagel et al., 2003), which is very effective at causing sustained depolarization and neuronal firing when the light is applied to ChR2-expressing somatodendritic regions of neurons (Boyden et al., 2005). However, when the continuous blue light is applied to ChR2-expressing presynaptic terminals, the initial presynaptic depolarization is very effective at triggering neurotransmitter release and postsynaptic actions but the ensuing sustained presynaptic depolarization does not correspond with sustained postsynaptic effects (Hormigo et al., 2019), possibly because sustained presynaptic depolarization is generally associated with presynaptic inhibition of neurotransmitter release (MacDermott et al., 1999). The distinct ability of blue light patterns to drive electrophysiological effects in networks of GABAergic neurons expressing ChR2 has been previously noted (Hormigo et al., 2016, 2019, 2021). For instance, activation of ChR2 expressed in somatodendritic regions of the recorded SNr neurons (Vgat-SNr-ChR2 mice) with continuous blue light is much more effective than trains (1-ms pulses) of blue light at evoking sustained neuronal firing in SNr neurons (Fig. 1*A*,*F*). Conversely, activation of ChR2 expressed in presynaptic GABAergic afferent fibers, originating in the striatum or Acb (Vgat-Str/Acb-ChR2 mice), with trains of blue light is much more effective than continuous blue light at evoking sustained IPSPs in the recorded SNr neuron (Fig. 1*B*). Therefore, when ChR2 is expressed in presynaptic terminals, continuous blue light produces an IPSP at light onset that adapts strongly and fades over time, while trains of blue light produce an IPSP on each pulse of the train which results in a robust sustained IPSP over the course of the light train (Hormigo et al., 2019, 2021).

Vgat-ChR2 mice (cross of Vgat-cre and ai32 mice) express ChR2 in both GABAergic SNr neurons and GABAergic afferent fibers reaching SNr, because Vgat-cre mice express cre recombinase in both GABAergic SNr and striatal neurons (Vong et al., 2011). Consequently, in these mice, blue light applied in SNr activates ChR2 in presynaptic GABAergic afferent fibers and in the somatodendritic region of SNr neurons, which generates simultaneous IPSPs and postsynaptic depolarization, respectively, in the same SNr neurons. Considering the differential effects of blue light patterns noted above when ChR2 is expressed in presynaptic fibers and in somatodendritic regions of SNr neurons, we reasoned that in Vgat-ChR2 mice SNr firing may be bidirectionally controlled (excited vs inhibited) by adjusting the pattern of blue light applied in SNr (continuous vs trains). To test this possibility, in the present study, we conducted whole-cell recordings from SNr neurons in acute slices of Vgat-ChR2 mice and applied different patterns of blue light in SNr. We found that the firing of the same SNr neuron was excited by continuous blue light but was inhibited by trains of blue light (Fig. 1*C–E*). The inhibition of SNr neuron firing is because of sustained IPSPs driven by each pulse in the light train (Fig. 1*D*, right panel). The resulting postsynaptic hyperpolarization and shunting keeps the brief postsynaptic ChR2 depolarization evoked by each 1 ms light pulse in the train from reaching firing threshold. In contrast, during continuous blue light, the IPSP only occurs at the onset of the light (Fig. 1*D*, middle panel) followed by a sustained depolarization, evoked by postsynaptic ChR2 activation, which drives robust SNr neuron firing. Thus, the repetitive blue light pulses in trains produce sustained inhibition of SNr neurons by maximizing IPSP recurrence and minimizing postsynaptic ChR2-evoked depolarization, while the continuous blue light produces sustained excitation because it has the opposite effects. In single neurons, the IPSP and postsynaptic depolarization are separable by adjusting the light power (Fig. 1*D*, left panel vs middle panel), the postsynaptic depolarization usually has lower threshold than the IPSPs, but this threshold is variable between neurons, perhaps reflecting different presynaptic and postsynaptic ChR2 expression levels. Since continuous blue light and trains of blue light applied in the SNr of Vgat-ChR2 mice have opposite effects on SNr neuronal firing, we next tested the effects of these light patterns on the behavior of mice exploring an open field.

### Bidirectional control of orienting behavior in Vgat-ChR2 mice

Consistent with the results of early work (Tarsy et al., 1975; Olianas et al., 1978; Di Chiara et al., 1979; Kilpatrick and Starr, 1981; Kilpatrick et al., 1982; Leigh et al., 1983), activating the direct and indirect striatonigral pathways can control movements by inhibiting and exciting SNr cells, respectively (Kravitz et al., 2010; Freeze et al., 2013; Hormigo et al., 2016). However, the differential effects of SNr excitation and inhibition on movement have only been studied by comparing different groups of animals. Our demonstration in slices that GABAergic SNr cell firing is excited or inhibited depending on the blue light pattern (continuous or trains) applied in the SNr provides an opportunity to determine whether orienting movement can be biased in opposite directions within the same Vgat-ChR2 animal. In the present study, orienting head movement is described versus the stimulated optical fiber (implanted in the brain) as ipsiversive (toward the side of the fiber) or contraversive (away from the side of the fiber). Whiskers (e.g., whisker tracking, EMG, air-puffs) are also described versus the side of the stimulated optical fiber as ipsilateral (on the side of the implanted fiber) or contralateral (on the opposite side).

**Figure 2.**
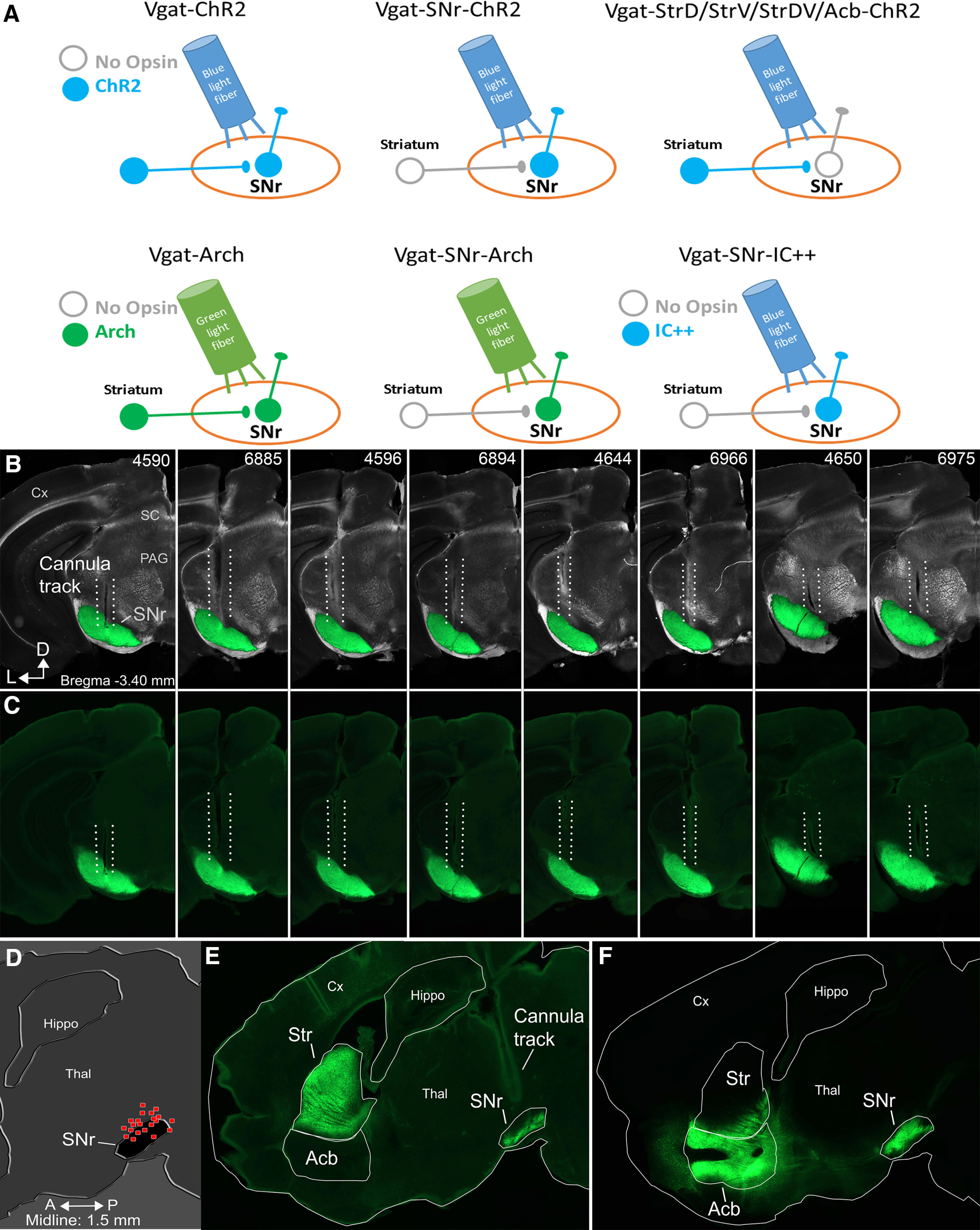
ChR2 expression and cannula placements. ***A***, Schematic of the different optogenetic groups of animals employed in the present study. Blue indicates blue light and expression of blue-sensitive opsins (ChR2 and IC++). Green indicates green light and expression of green-sensitive opsin (Arch). As noted, opsin expression occurs in GABAergic afferents that reach the SNr from neurons located in different parts of the striatum (including StrD and/or StrV, or Acb) and/or in GABAergic neurons located in the SNr. Light is always applied in the SNr. ***B***, ***C***, Coronal sections at the level of the midbrain from eight Vgat-ChR2 mice showing the robust expression of ChR2 circumscribed to the SNr and the optical fiber tract by which blue light was applied during behavior. The upper panels (***B***) blend a dark-field image of the section with the green channel of the eYFP fluorescence image. The lower panels (***C***) show the fluorescence image alone. The top right numbers are animal IDs. ***D***, Reconstruction of optical fiber track endings in the SNr for all mice in the study. ***E***, Location of AAV injections in the striatum and corresponding projections in the SNr of Vgat-StrDV-ChR2 mice. ***F***, Location of AAV injections in the Acb and corresponding projections in the SNr of Vgat-Acb-ChR2 mice.

Figure 2*A* shows a schematic of the different optogenetic groups used in the study. We implanted Vgat-ChR2 mice with an optical fiber in the SNr (Fig. 2*B*,*C* shows the tracks of optical fibers from eight SNr sites in these mice, and Fig. 2*D* shows a schematic of the location of optical fiber endings in the SNr from all the animals in the study) and subsequently (more than one week later) placed the mice in an open field arena while we tracked (100 FPS) head movements with two color LED markers located in the head implant over the nose and between the ears (36 sessions in six mice). As the animals explored the arena, we interleaved continuous (2 s; Cont) blue light and trains (1-ms pulses at 40–66 Hz; 2 s; train) of blue light unilaterally in the SNr of Vgat-ChR2 mice. This allowed comparing the effect of Cont and train blue light within the same mice and sessions on head direction bias (cumulative sum of the change in head angle in the ipsiversive or contraversive direction with respect to the side of the stimulated optical fiber in SNr) and speed (instantaneous Δ speed from light onset). Note that Figure 5 shows a summary of population orienting data from the different optogenetic groups.

**Figure 3.**
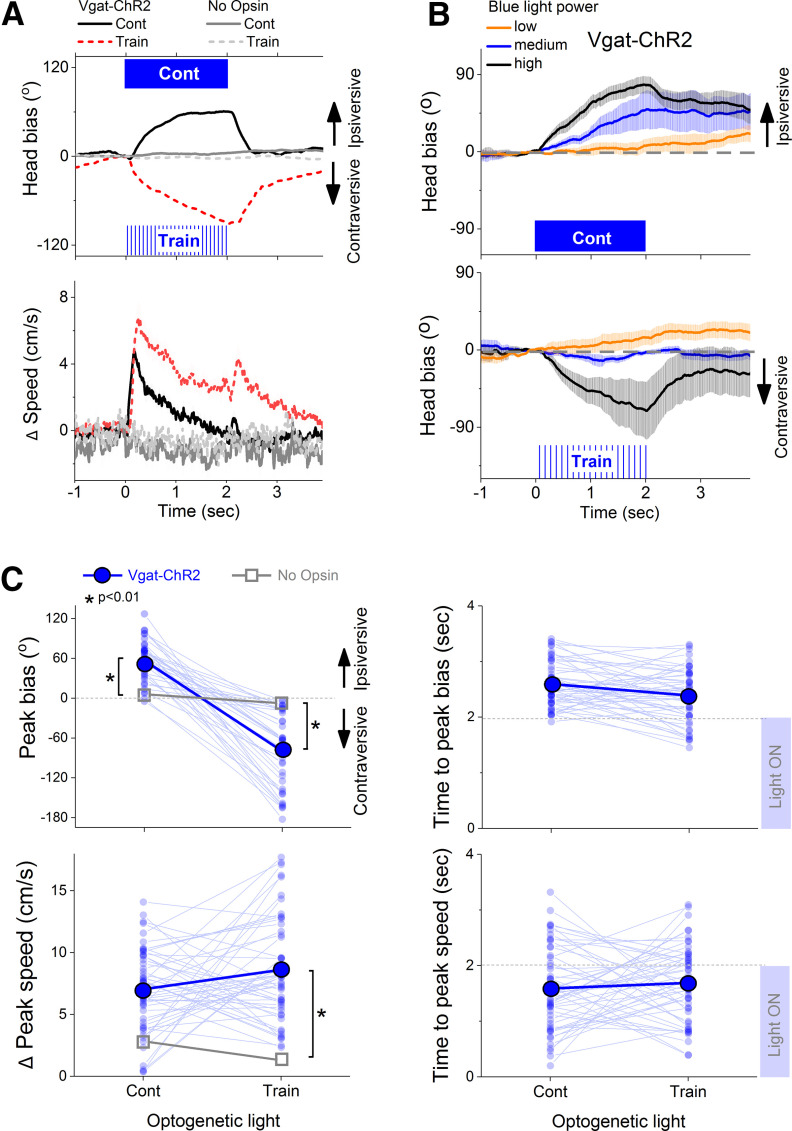
Effect of trains or continuous blue light in the SNr of Vgat-ChR2 mice on head orienting movements (head bias) during exploration of an open field. ***A***, upper, Head bias evoked by blue light in the ipsiversive (positive) or contraversive (negative) direction versus the side of the brain where the stimulated optical fiber is implanted. The traces overlay the effects in Vgat-ChR2 mice and in No Opsin mice. Lower, Change in speed from blue light onset associated with the head bias. ***B***, Effect of continuous blue light (upper) or trains of blue light (lower) at different powers (0.7, 1.6, 3.0 mW) on head bias. ***C***, Population measures of peak head bias, change in peak speed and times to these peaks (if the peaks exist) for Vgat-ChR2 and No Opsin mice. Asterisks show significant differences between the two groups of mice.

Remarkably, within the same animal in the same session, Cont blue light in the SNr produced an ipsiversive direction bias while train blue light produced a contraversive direction bias (Fig. 3*A*). Thus, the direction of the head movement depended on whether the blue light was exciting (Cont) or inhibiting (train) SNr cells, and the effects increased with light power (Fig. 3*B*), unless otherwise indicated, hereafter (for simplicity) we combined the different light powers tested after determining that they produced effects in the same direction. Importantly, application of these same light patterns in the SNr of mice that did not express ChR2 (No Opsin; 24 sessions in three mice) did not affect direction bias or speed during exploration of the open field (Fig. 3*A*). To determine whether the effects on direction bias and speed were significant, we compared the opsin-expressing mice to the No Opsin mice (Fig. 3*C*). Statistical analyses involved both ANOVAs (followed by Tukey’s) and bootstrapping comparisons (the bootstrapping *p* value is shown in brackets [*p* =]).

Overall, Cont blue light in the SNr evoked a 56.7 ± 5.3° (mean ± SEM) ipsiversive bias (Tukey’s *t*_(195)_ = 7.53 *p* < 0.0001 [bootstrapping *p* < 0.0001]; Vgat-ChR2 vs No Opsin) that peaked at 2.17 ± 0.05 s and reached a peak speed of 10.38 ± 0.96 cm/s (Tukey’s *t*_(195)_ = 4.9 *p* = 0.04 [*p* = 0.012]; Vgat-ChR2 vs No Opsin) at 1.53 ± 0.1 s after light onset. In contrast, train blue light in the SNr evoked a 79.9 ± 9.6° contraversive bias (Tukey’s *t*_(195)_ = 6.37 *p* < 0.0001 [*p* = 0.001]; Vgat-ChR2 vs No Opsin) that peaked at 2.07 ± 0.07 s and reached a peak speed of 12.63 ± 1.36 cm/s (Tukey’s *t*_(195)_ = 6.06 *p* = 0.0022 [*p* < 0.0001]; Vgat-ChR2 vs No Opsin) at 1.63 ± 0.09 s after light onset (Fig. 3*C*). Comparison of the effects between Cont and train blue light within the same Vgat-ChR2 mice revealed that direction bias was significantly different (Tukey’s *t*_(226)_ = 16.06 *p* < 0.0001 [*p* < 0.0001]; Cont vs train), but peak speed was not (Tukey’s *t*_(226)_ = 2.41 *p* = 0.94 [*p* = 0.546]; Cont vs train). Thus, the mice move in opposite directions with Cont and train but reach a similar peak speed. Comparison of the absolute movement bias (ignoring the direction) was not significantly different (Tukey’s *t*_(26)_ = 2.19 *p* = 0.13 [*p* = 0.24]; Cont vs train). Thus, Cont and train blue light applied in the SNr of Vgat-ChR2 mice drives head movement orienting in opposite directions but similar amplitudes.

### SNr excitation produces only ipsiversive orienting

**Figure 4.**
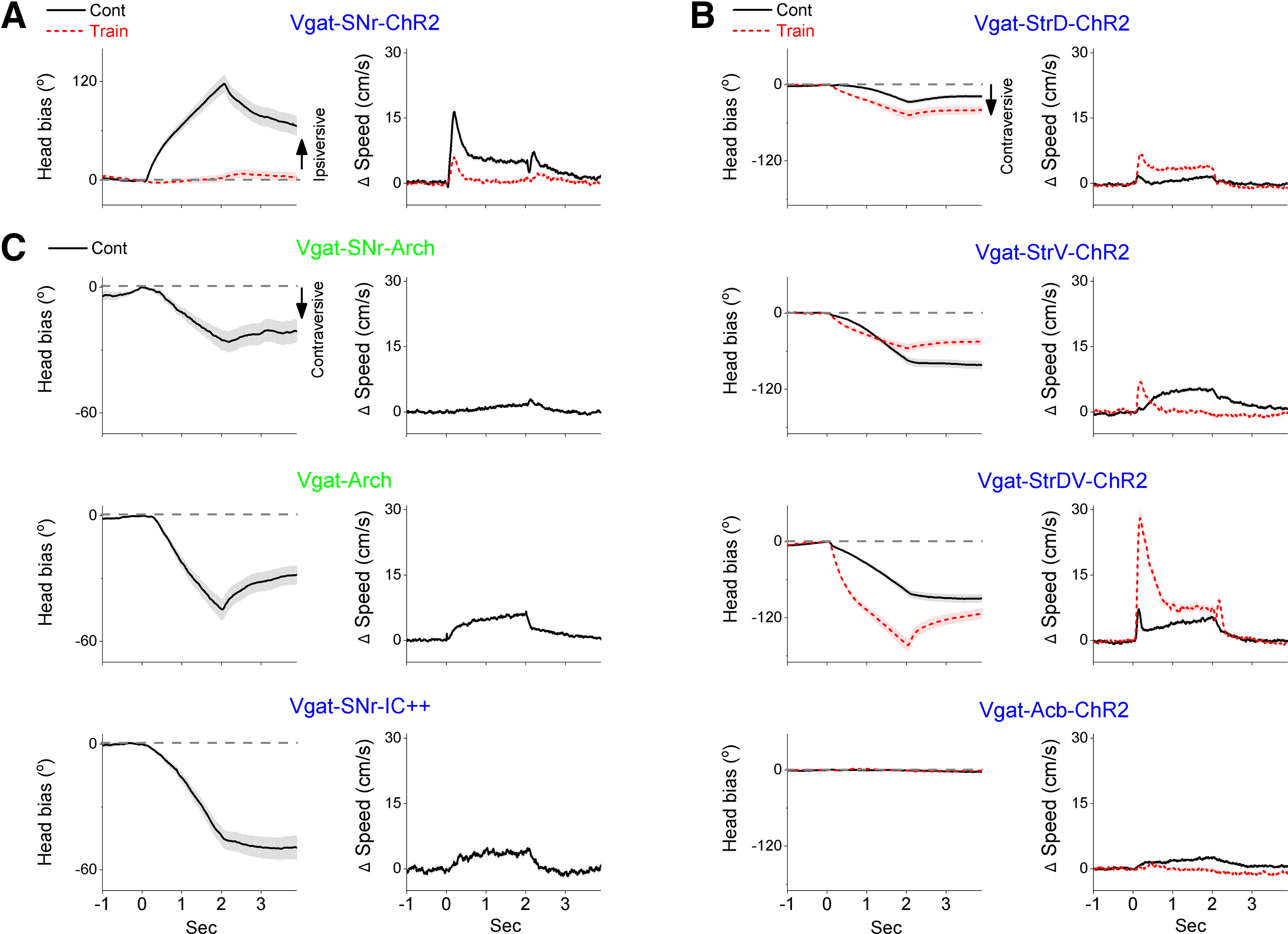
Effect of trains or continuous light on head orienting movements during exploration of an open field in different mice that express opsin only in GABAergic SNr cells or in afferent fibers. Panels show population mean ± SEM. ***A***, Effect of exciting GABAergic SNr neurons using blue light in Vgat-SNr-ChR2 mice on head bias and change in speed. ***B***, Effect of inhibiting SNr neurons by exciting GABAergic afferent fibers in SNr using blue light in Vgat-StrD-ChR2, Vgat-StrV-ChR2, Vgat-StrDV-ChR2, and Vgat-Acb-ChR2 mice on head bias and change in speed. ***C***, Effect of inhibiting GABAergic SNr neurons using green light in Vgat-SNr-Arch and Vgat-Arch mice, and blue light in Vgat-SNr-IC++ mice on head bias and change in speed.

Vgat-ChR2 mice express ChR2 in both GABAergic SNr neurons (efferents) and afferents. To dissociate the effects of blue light applied in the SNr of Vgat-ChR2 mice, we expressed ChR2 selectively in GABAergic SNr neurons or in GABAergic afferents reaching the SNr. In Vgat-SNr-ChR2 mice, an AAV was injected in the SNr to selectively express ChR2 in GABAergic SNr neurons. In these mice (20 sessions in three mice), both Cont and train blue light excite SNr neurons, although Cont produces much stronger excitation (Fig. 1*A*,*F*; Hormigo et al., 2016). We found that Cont blue light (Fig. 4*A*) applied in the SNr evoked a 118.96 ± 14.14° ipsiversive bias (Tukey’s *t*_(195)_ = 5.4, *p* = 0.012 [*p* < 0.0001]; Vgat-SNr-ChR2 vs No Opsin) that peaked at 2.44 ± 0.04 s and reached a peak speed of 16.12 ± 0.82 cm/s (Tukey’s *t*_(195)_ = 5.37, *p* = 0.016 [*p* = 0.0093]; Vgat-SNr-ChR2 vs No Opsin) at 1.44 ± 0.07 s after light onset. In contrast, train blue light (Fig. 4*A*) did not produce a significant direction bias (Tukey’s *t*_(195)_ = 1.42, *p* = 0.99 [*p* = 0.955]; Vgat-SNr-ChR2 vs No Opsin) or a change in peak speed (Tukey’s *t*_(195)_ = 0.46, *p* = 0.99 [*p* = 0.938]; Vgat-SNr-ChR2 vs No Opsin). Train blue light tended to move Vgat-SNr-ChR2 mice ipsiversively (23.63 ± 13.58°) but this was not significantly different compared with No Opsin mice. Consequently, in Vgat-SNr-ChR2 mice, Cont blue light produces a much stronger ipsiversive bias (Tukey’s *t*_(226)_ = 3.89 *p* < 0.0001 [*p* = 0.002]; Cont vs train) and peak speed (Tukey’s *t*_(226)_ = 4.69 *p* < 0.0001 [*p* = 0.007]; Cont vs train) compared with train blue light. Thus, Cont excitation of GABAergic SNr neurons drives a strong ipsiversive head orienting bias while train excitation has little effect, albeit in the same direction.

### SNr inhibition produces only contraversive orienting

**Figure 5.**
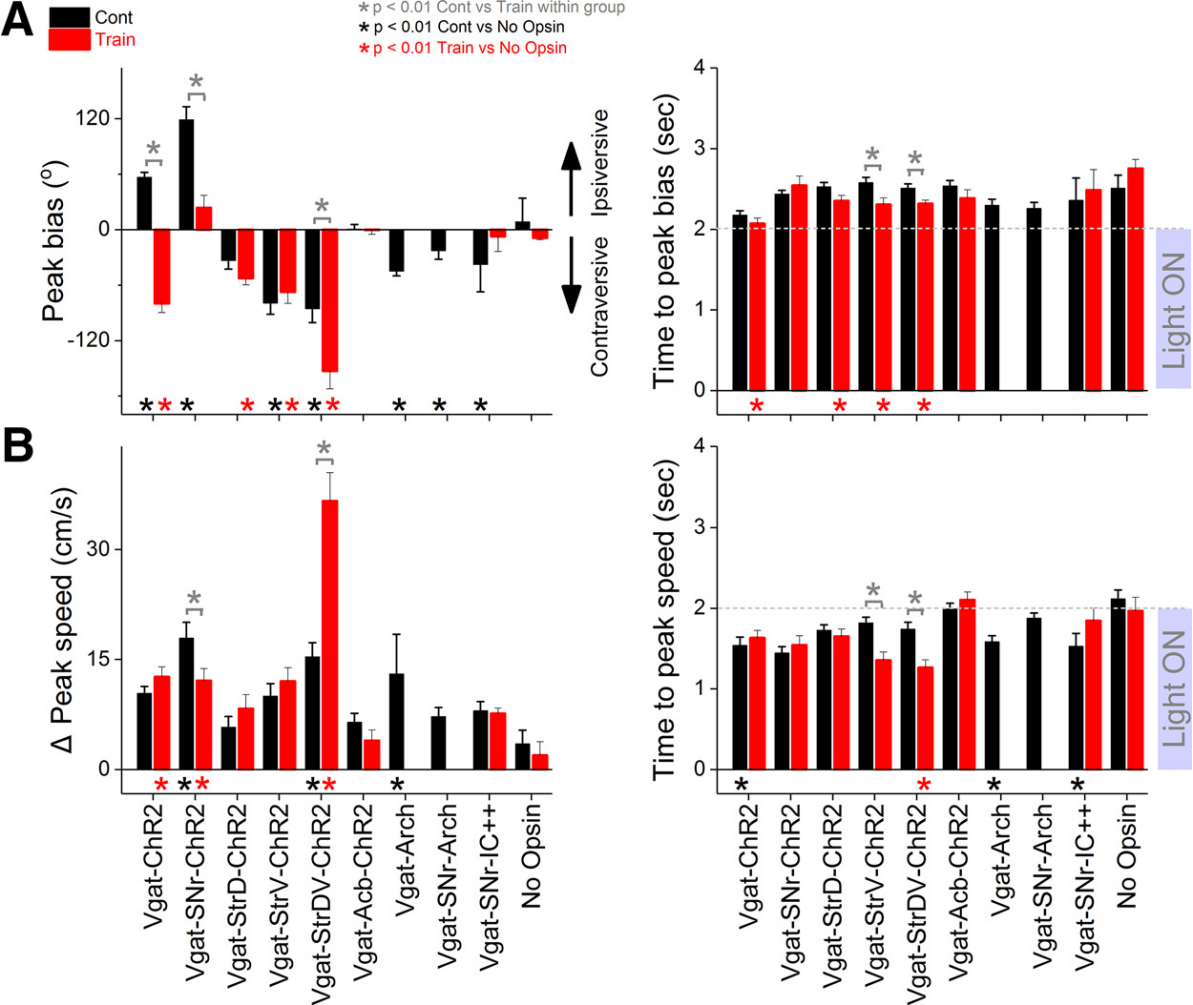
Population data of peak head bias, change in peak speed, and times to these peaks (if they exist) for the different groups in our study. ***A***, Peak head bias and time to peak for the different groups. Gray asterisks on brackets denote significant differences between trains and continuous blue light within a group. Black or red asterisks at the bottom denote significant differences versus No Opsin mice. ***B***, Same as ***A*** but for change in peak speed and time to peak speed.

We then tested the effect of Cont and train blue light in the SNr of mice that express ChR2 in GABAergic afferents reaching the SNr. There are several sources of GABAergic afferents to the SNr (which express cre in vgat-mice; Vong et al., 2011), including major projections from striatum and Acb; other sources of GABAergic fibers (e.g., globus pallidus) were not explored. Mice were injected with an AAV to express ChR2 in GABAergic cells of the StrD (Vgat-StrD-ChR2), the StrV (Vgat-StrV-ChR2), the dorsal and ventral striatum (Vgat-StrDV-ChR2; see Fig. 2*E*), or the Acb (Vgat-Acb-ChR2; see Fig. 2*F*). In these mice, both Cont and train blue light applied in the SNr inhibited SNr cells, although train is much more effective than Cont at sustaining inhibition (Fig. 1*B*,*F*; Hormigo et al., 2021). In the three groups with GABAergic fibers originating in the striatum, both Cont and train blue light in the SNr evoked a contraversive bias (Fig. 4*B*) that was strongest for the group that engaged striatonigral fibers originating in both the dorsal and ventral striatum (i.e., Vgat-StrDV-ChR2). Thus, in Vgat-StrDV-ChR2 (36 sessions in three mice), Cont evoked a 85.61 ± 14.9° contraversive bias (Tukey’s *t*_(195)_ = 8.88, *p* < 0.0001 [*p* < 0.0001]; Vgat-StrDV-ChR2 vs No Opsin) that peaked at 2.51 ± 0.05 s and reached a peak speed of 15.38 ± 1.92 cm/s (Tukey’s *t*_(195)_ = 6.11, *p* = 0.0019 [*p* = 0.0021]; Vgat-StrDV-ChR2 vs No Opsin) at 1.74 ± 0.09 s after light onset. Train evoked a stronger 153 ± 19.19° contraversive bias (Tukey’s *t*_(195)_ = 12.16, *p* < 0.0001 [*p* < 0.0001]; Vgat-StrDV-ChR2 vs No Opsin) that peaked at 2.31 ± 0.04 s and reached a peak speed of 36.55 ± 3.88 cm/s (Tukey’s *t*_(195)_ = 15.85 *p* < 0.0001 [*p* < 0.0001]; Vgat-StrDV-ChR2 vs No Opsin) at 1.26 ± 0.1 s after light onset. In these animals, train blue light produced a stronger contraversive bias (Tukey’s *t*_(226)_ = 4.60, *p* < 0.0001 [*p* = 0.0046]; Cont vs train) and peak speed (*t*_(226)_ = 12.20, *p* < 0.0001 [*p* < 0.0001]; Cont vs train) compared with Cont blue light. Similar findings occurred when ChR2 expression was limited to either the StrD (Vgat-StrD-ChR2, 45 sessions in 5 mice) or the StrV (Vgat-StrV-ChR2, 34 sessions in three mice), albeit the strength of these effects was smaller compared with simultaneously exciting fibers from both striatal regions (Fig. 4*B*). Thus, excitation of afferent GABAergic SNr fibers originating in the striatum with train and Cont, which inhibits SNr neurons, produces a head orienting bias that is opposite to exciting SNr cells (Fig. 5). Moreover, consistent with the ability of train to sustain IPSPs in SNr neurons, when GABAergic afferent fibers are excited, train blue light tends to produce a stronger contraversive orienting bias than Cont blue light.

Interestingly, when the GABAergic fibers originated in the Acb (Vgat-Acb-ChR2, 46 sessions in three mice) neither Cont nor train blue light in the SNr had any significant effect on movement (Fig. 4*B*). Both Cont (0.63 ± 4.99°; Tukey’s *t*_(195)_ = 1.54, *p* = 0.99 [*p* = 0.772]; Vgat-Acb-ChR2 vs No Opsin) and train (0.41 ± 4.79°; Tukey’s *t*_(195)_ = 0.49 *p* = 1 [*p* = 0.982]; Vgat-Acb-ChR2 vs No Opsin) evoked negligible movement that did not differ in direction bias (Tukey’s *t*_(226)_ = 0.32, *p* = 1 [*p* = 0.559]; Cont vs train) or peak speed (Tukey’s *t*_(226)_ = 0.41, *p* = 1 [*p* = 0.426]; Cont vs train).

Finally, we also tested the effect of directly inhibiting GABAergic SNr cells using Arch (Cont green light applied in SNr) or IC++ (Cont blue light applied in SNr). In these mice, SNr cells are inhibited without exciting afferent fibers reaching SNr, which could inhibit other nuclei via fiber collaterals and complicate interpretation of the effects mediated by SNr. These opsins are effective at inhibiting SNr cells both in slices and in vivo (Hormigo et al., 2016, 2021). Here, we employed three groups of mice. Vgat-Arch mice (cross of Vgat and ai40D) express ArchT broadly in GABAergic cells, including SNr cells. Vgat-SNr-Arch mice were injected an AAV in the SNr to selectively express eArch3.0 in GABAergic SNr cells. Vgat-SNr-IC++ mice were injected an AAV in the SNr to selectively express IC++ in GABAergic SNr cells.

In all these groups, Cont produced a contraversive bias (Fig. 4*C*). In Vgat-Arch mice (28 sessions in 4 mice), Cont green light evoked a 45.06 ± 4.97° contraversive bias (Tukey’s *t*_(195)_ = 21.2, *p* < 0.0001 [*p* < 0.0001], Vgat-Arch vs No Opsin) that peaked at 2.3 ± 0.07 s and reached a peak speed of 13.01 ± 5.42 cm/s (Tukey’s *t*_(195)_ = 10.26, *p* < 0.0001 [*p* = 0.0013], Vgat-Arch vs No Opsin) at 1.58 ± 0.07 s from light onset. In Vgat-SNr-Arch mice (36 sessions in 5 mice), Cont green light evoked a 22.76 ± 9.22° contraversive bias (Tukey’s *t*_(195)_ = 7.35, *p* = 0.007 [*p* < 0.0001], Vgat-SNr-Arch vs No Opsin) that peaked at 2.26 ± 0.07 s but the peak speed (7.2 ± 1.24 cm/s) of this movement did not differ compared with No Opsin mice (Tukey’s *t*_(195)_ = 2.38, *p* = 0.75 [*p* = 0.208], Vgat-SNr-Arch vs No Opsin). In Vgat-SNr-IC++ mice (18 sessions in 5 mice), Cont blue light evoked a 37.75 ± 29.62° contraversive bias (Tukey’s *t*_(195)_ = 5.72, *p* = 0.0068 [*p* < 0.0001]; Vgat-SNr-IC++ vs No Opsin) that peaked at 2.36 ± 0.28 s but the peak speed of the movement (8 ± 1.25 cm/s) did not differ compared with No Opsin mice (Tukey’s *t*_(195)_ = 2.83, *p* = 0.82 [*p* = 0.244]; Vgat-SNr-IC++ vs No Opsin). Thus, similar to inhibiting SNr cells by exciting GABAergic afferents, direct inhibition of GABAergic SNr neurons (with Arch or IC++) drives contraversive orienting at about the same or slightly higher speed than ongoing movement.

### SNr-driven orienting and whisker positioning

As animals move to explore the environment, they orient their sensors in the appropriate direction. For example, during gaze shifts, the eyes can lead the head in making an initial saccade to a new spatial location (Gandhi and Katnani, 2011). Similarly, whiskers anticipate head movements by pointing in the direction of the head movement, creating bilateral asymmetries in whisker positions between both sides of the face (Towal and Hartmann, 2006). We determined whether the bidirectional head orienting evoked by different patterns of blue light in the SNr of Vgat-ChR2 mice was associated with changes in whisker position and whether those changes are congruent when the head moves in opposing directions.

**Figure 6.**
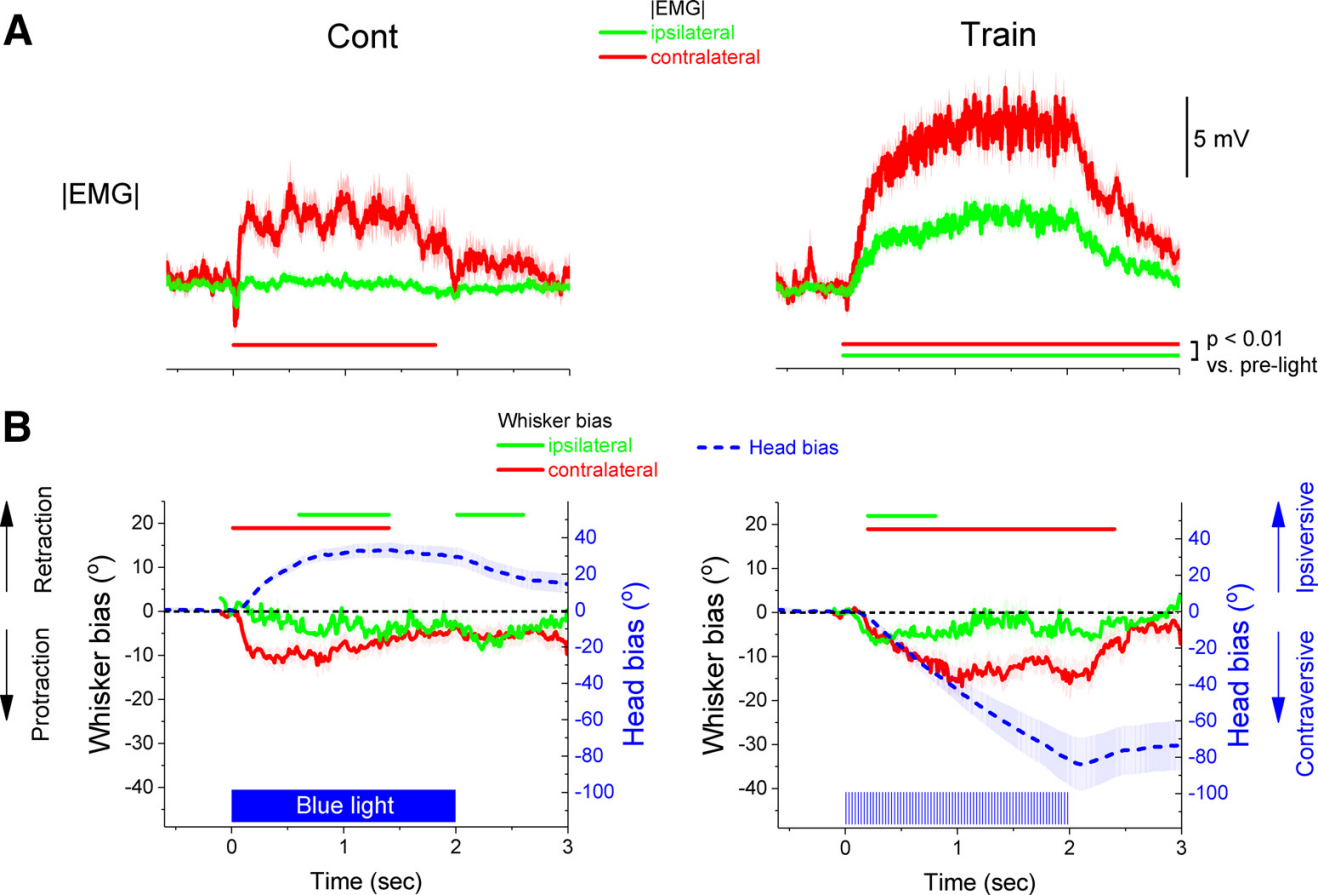
Effect of blue light on whisker position during head bias evoked by blue light in Vgat-ChR2 mice. ***A***, EMG recorded from the ipsilateral (green) and contralateral (red) whisker pads. The left panels show the effect of continuous blue light (Cont) while the right panels show the effect of trains of blue light. Panels show population mean ± SEM for all the sessions. ***B***, Shows the position of the whiskers versus the head (whisker bias) evoked by the blue light in the same sessions as ***A***. The green trace tracks the ipsilateral whiskers while the red trace tracks the contralateral whiskers. Overlaid in blue is the head bias. The green and red color lines parallel to the *x*-axis denote the period when the whisker bias traces are significantly different compared with the prelight period based on a bootstrap comparison.

We tracked whisker position in head centered coordinates and measured whisker pad EMG bilaterally as optogenetic blue light (Cont vs train) was delivered unilaterally in the SNr of mice exploring the arena (20 sessions in four mice; Fig. 6). To define the portion of the tracked whisker movements that were significantly affected by the optogenetic light patterns, we used a bootstrap comparison versus a baseline period before the light (200-ms windows; significant effects are highlighted per each trace in bright colors; Fig. 6). Cont blue light (Fig. 6*B*, left) produced ipsiversive head orienting accompanied by protraction of the contralateral whiskers but minimal change in the position of the ipsilateral whiskers versus the head. Accordingly, the contralateral EMG (Fig. 6*A*, left) showed a sharp increase associated with the whisker protraction while the ipsilateral EMG was flat (apart from a brief startle observed on both sides at light onset). Thus, the contralateral whiskers actively protract and point in the direction of the ipsiversive head movement caused by exciting SNr with Cont blue light.

In contrast, train blue light produced contraversive head orienting that was associated with protraction of both the contralateral and ipsilateral whiskers (Fig. 6*B*, right). The EMG activated bilaterally indicating active muscle engagement on both sides (Fig. 6*A*, right). However, the changes on the contralateral side were more prominent than the changes on the ipsilateral side. For instance, as the head moved contraversively, the contralateral whiskers remained protracted while the ipsilateral whiskers returned to their original position. Thus, during contraversive orienting caused by inhibiting SNr, the contralateral whiskers point in the direction away from the head movement while the ipsilateral whiskers only briefly point in the direction of the head movement.

In conclusion, head orienting caused by modulating SNr is associated with whisker positioning that is not congruent with the direction of the orienting movement; both ipsiversive (Cont) and contraversive (train) head orienting produced protractions of the contralateral whiskers. If head orienting in both directions was equivalent, the whiskers should position themselves according to the orienting direction, pointing in the direction of the head movement. Instead, the contralateral whiskers protract for both ipsiversive and contraversive head movements pointing in the direction of the movement only during ipsiversive orienting. This suggests that orienting biases caused by exciting (Cont) or inhibiting (train) SNr may have different behavioral significance for the animal.

### SNr-driven orienting and behavioral significance

Orienting movements adapt to changing environmental stimuli. For instance, animals rapidly adjust orienting direction when there is an obstacle or a penalty for moving in that direction. We next determined whether application of an external sensory stimulus (air puff) to the whiskers, which behaving mice find annoying, affects head orienting biases caused by SNr excitation or inhibition. The air puff was adjusted to produce minimal head orienting; only a slight turn away from the side of the air puff nozzle. We reasoned that the annoying air puff should antagonize an approaching or exploratory movement (toward the air-puff nozzle side) but enhance a retracting or defensive movement (away from the air-puff nozzle side). Thus, if the head movements caused by Cont (ipsiversive) and train (contraversive) were differentially affected by an air puff it would indicate that these movements have a different behavioral significance.

**Figure 7.**
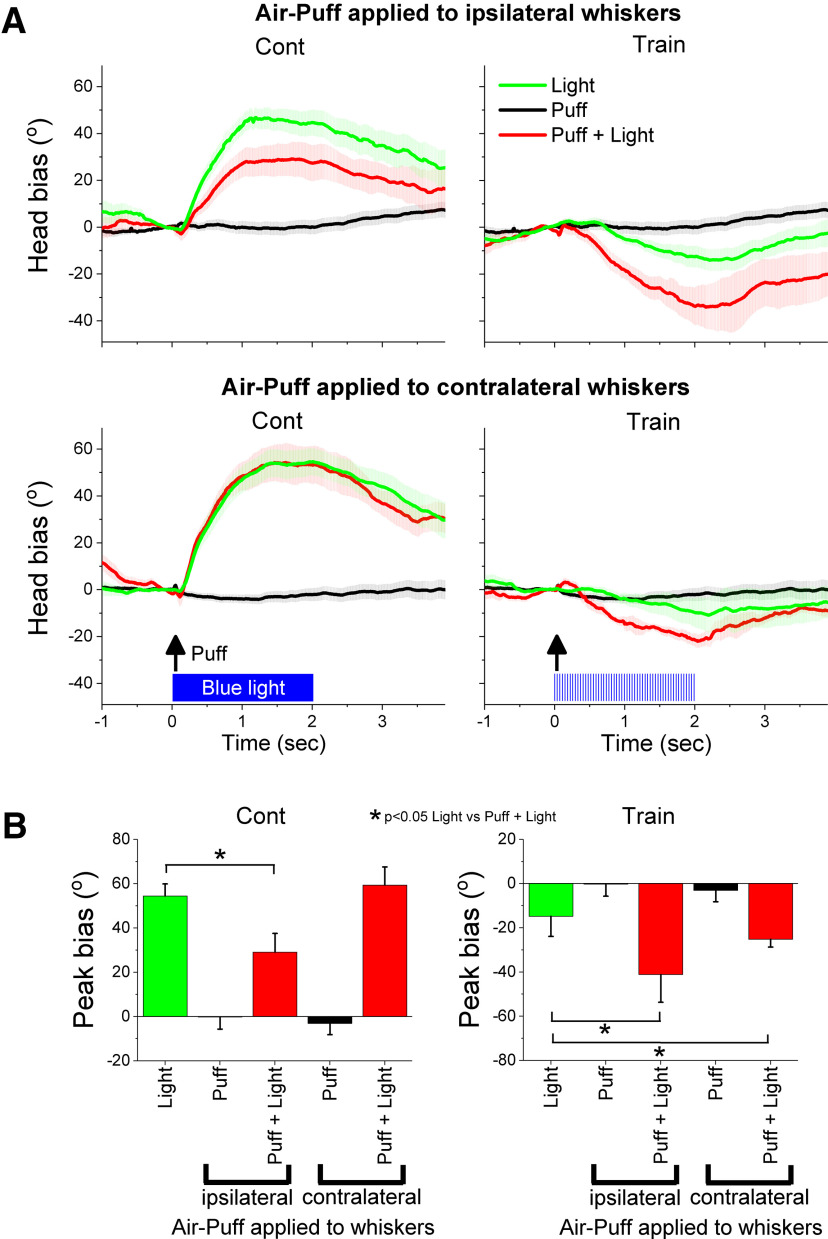
Effect of an air puff applied to the ipsilateral or contralateral whiskers on head bias evoked by blue light in Vgat-ChR2 mice. ***A***, Air-puff applied to the ipsilateral (upper) and contralateral (lower) whiskers on head bias evoked by continuous or trains of blue light. The traces show the effect of light alone, an air-puff alone, or the light and air-puff delivered together. Panels show population mean ± SEM for all the sessions. ***B***, Population measures of peak head bias for the different conditions shown in ***A***. The asterisks denote differences between the light alone versus the light and air-puff applied together.

In freely behaving Vgat-ChR2 mice (20 sessions in four mice), an air puff was delivered to the ipsilateral or contralateral whiskers in relation to train and Cont in SNr. The air-puffs were delivered through a hand-held tube positioned in front of the whiskers on one side of the face, to deflect the whiskers on that side. Trials of different types were randomly delivered throughout the same session. As noted, the air puffs applied alone produced very slight (negligible) head orienting (Fig. 7). Cont or train blue light in SNr alone produced ipsiversive and contraversive head orienting, respectively. However, combining the air puffs and the blue light in SNr led to different effects depending on the optogenetic stimulus. An ipsilateral air puff antagonized the ipsiversive head orienting caused by exciting SNr with Cont (Tukey’s *t*_(16)_ = 4.99, *p* = 0.02 [*p* = 0.005]), while a contralateral air-puff had no effect on this head movement (Tukey’s *t*_(16)_ = 0.97, *p* = 0.95 [*p* = 0.53]). In contrast, both ipsilateral (Tukey’s *t*_(16)_ = 4.84, *p* = 0.024 [*p* = 0.044]) and contralateral (Tukey’s *t*_(16)_ = 4.59, *p* = 0.035 [*p* = 0.023]) air puffs enhanced the contraversive head orienting caused by inhibiting SNr with train. The fact that the air-puffs (especially the ipsilateral air-puff) enhanced contraversive orienting suggests that the contraversive movement caused by inhibiting SNr has a retracting or defensive (move away) behavioral significance. On the other hand, the fact that the ipsilateral air-puff suppressed ipsiversive orienting suggests that the ipsiversive movement caused by exciting SNr has an approaching or exploratory (move toward) behavioral significance. In other words, SNr inhibition may signal the animal to move away, while SNr excitation may signal the animal to move toward.

### Bidirectional control of goal-directed behavior by SNr

The previous results show that different patterns of blue light (Cont vs train) in the same Vgat-ChR2 mice produce opposite effects on the firing of SNr neurons (excitation vs inhibition) and head orienting biases (ipsiversive vs contraversive) of distinct behavioral significance during active exploration of an arena. In particular, unilateral inhibition of SNr neurons causes contraversive orienting movement that may have a moving away significance. Previous studies have shown that signaled active avoidance, a goal-directed behavior during which mice must move away to avoid an aversive US, depends on the firing of GABAergic SNr neurons (Hormigo et al., 2016, 2019, 2021), excitation of SNr neurons in Vgat-SNr-ChR2 mice blocks signaled active avoidance, while inhibition of these neurons in Vgat-SNr-Arch drives active avoidance in the absence of an external signal (CS). The bidirectional control of SNr firing in Vgat-ChR2 mice provides a unique opportunity to test the effect of exciting and inhibiting SNr neurons on signaled active avoidance within the same animal. We used Vgat-ChR2 mice to determine whether blue light patterns that excite or inhibit SNr firing can control the ability of the same mice to perform a goal-directed behavior (signaled active avoidance).

Mice trained in signaled active avoidance and implanted with optical fiber cannulas bilaterally in the SNr underwent daily sessions in which three different trial types (ACS, ACS+LCS, and LCS alone trials) were presented randomly. ACS trials are control trials in which an auditory tone (ACS; ∼85 dB, 8 kHz) is presented alone during the avoidance interval (7 s), which is followed by a US (white noise and footshock) if the animal does not avoid. A successful avoidance response occurs when the animal shuttles by moving to the adjacent compartment in a shuttle box during the avoidance interval. If an avoidance response does not occur, the US drives an escape response consisting of a rapid shuttle to the adjacent compartment. In LCS alone trials, the optogenetic stimulation (1.5 mW) replaces the ACS to determine whether the LCS can drive avoidance on its own (in the absence of the ACS). In ACS+LCS trials, the optogenetic stimulation (1.5 mW) occurs simultaneously with the ACS and persists during presentation of the US if the animal does not avoid; these trials determine whether the optogenetic stimulation affects the ability of the ACS to drive avoidance responses. Mice are free to shuttle (intertrial crossings) during the random intertrial interval (25- to 45-s duration), which is devoid of optogenetic stimulation or any consequence for shuttling. Optogenetic stimulation during ACS+LCS or LCS alone trials consisted of continuous blue light or trains (10, 20, or 40 Hz; 1-ms pulses) of blue light at the same powers used during orienting experiments applied bilaterally in the SNr (light offset occurs when the animal avoids or escapes). In all analyses, we compared ACS trials (i.e., control trials) versus ACS+LCS or LCS alone trials within the same sessions; ACS+LCS and LCS alone trials were tested in different sessions.

**Figure 8.**
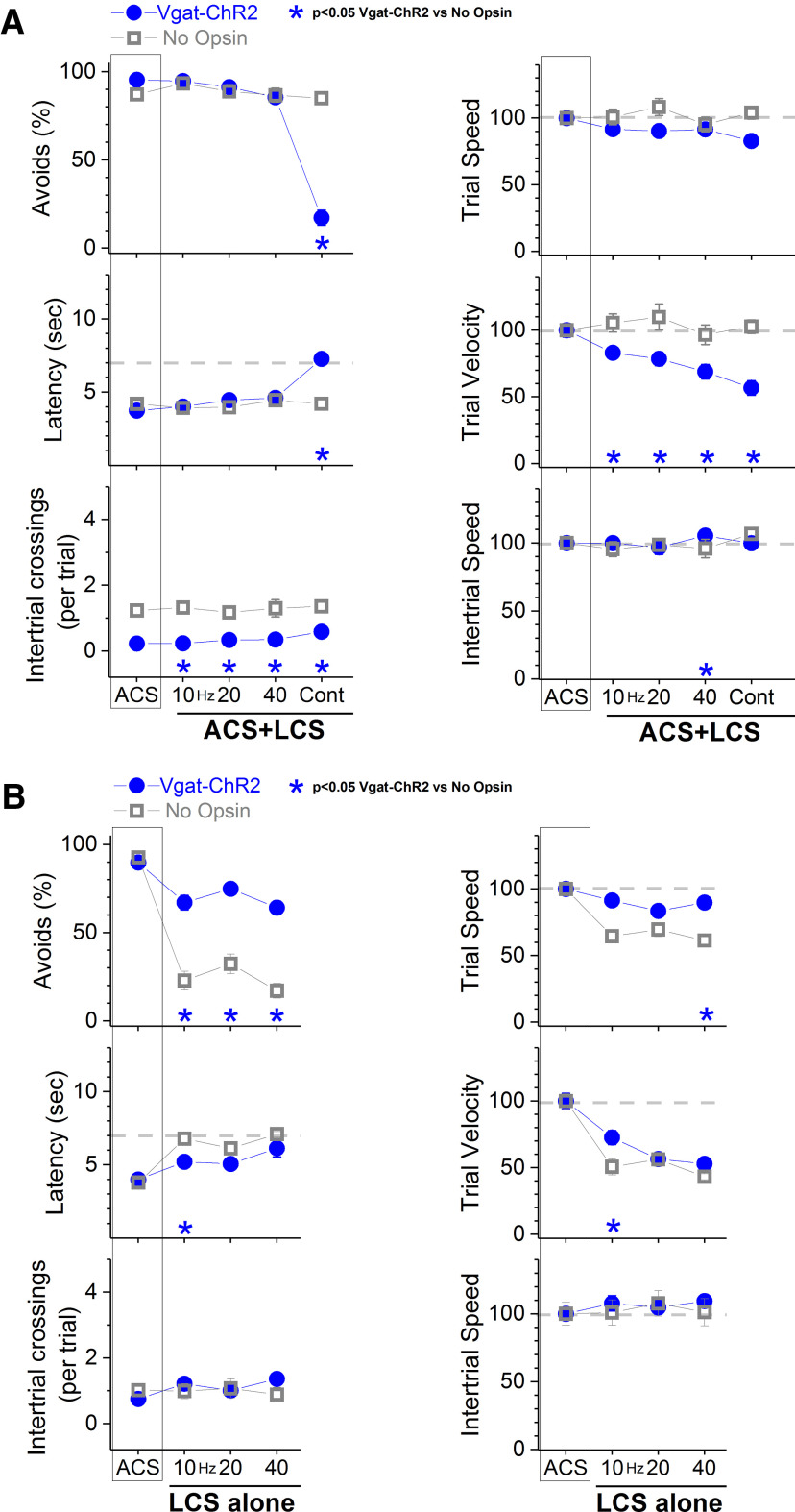
Effects of blue light in the SNr of Vgat-ChR2 mice on signaled active avoidance. ***A***, Effect of continuous or trains of blue light in the SNr of Vgat-ChR2 mice and No Opsin mice on ACS+LCS trials, which test the effect of the light pattern on the ability to perform signaled active avoidance to an ACS. Note that continuous blue light impaired active avoidance in Vgat-ChR2 mice but not in No Opsin mice. The right panels show trial speed, trial velocity, and intertrial speed for the data in the left panels. Asterisks denote blue light patterns that were significantly different between the two groups. ***B***, Effect of trains of blue light, which inhibit SNr in Vgat-ChR2 mice (but not in No Opsin mice), on ACS alone trials. In ACS alone trials, the SNr inhibition substitutes the regular external ACS to determine whether it has the ability to serve as a CS and trigger active avoidance responses. The right panels show trial speed, trial velocity, and intertrial speed for the data in the left panels. Asterisks denote blue light patterns that were significantly different between the two groups.

In ACS+LCS trial sessions (Fig. 8*A*, closed blue circles), continuous blue light, which increases GABAergic SNr firing in Vgat-ChR2 mice, blocked avoidance responses during presentation of the ACS, while trains of blue light had negligible effects (ACS+LCS, *n* = 16 sessions in 4 mice). Despite abolishing avoidance responses with continuous blue light, the animals had little impairment escaping the US, as noted by the fast escape latencies (occurring at ∼7 s, when the US starts). Application of the same procedures to No Opsin mice (*n* = 20 sessions in 4 mice) had no effect on active avoidance (Fig. 8*A*, open gray squares). Comparison between the two groups of animals revealed that the percentage of avoidance responses during Cont were significantly lower in Vgat-ChR2 mice compared with No Opsin mice (Tukey’s *t*_(60)_ = 20.9, *p* < 0.0001 [*p* < 0.0001]), but not during train (10–40 Hz). Thus, the effects observed in Vgat-ChR2 mice are because of activation of the opsin and not because of spurious effects of the blue light. In Vgat-ChR2 mice, the blue light inhibited trial velocity (movement in the correct direction to avoid) but not trial speed (overall movement in any direction) during ACS+LCS trials as a function of light frequency, with maximal suppression of trial velocity during Cont (which also suppresses avoidance responses). One possibility is that during trains (increasing in frequency), the SNr inhibition distracts the animals, which postpone the movement in the direction to avoid (they explore in different directions before avoiding). In addition, the No Opsin mice had more overall intertrial crossings (i.e., spurious exploratory crossings) than the Vgat-ChR2 mice. This may be a consequence of the fact that the Vgat-ChR2 mice are shocked during Cont (when they fail to avoid), which could make mice more cautious; as reflected in a reduction of spurious exploratory crossings.

In LCS alone trial sessions (Fig. 8*B*), we tested the ability of the blue light trains to serve as an effective CS to drive avoidance responses. We found that LCS alone trials (10–40 Hz) of blue light effectively drove active avoidance responses in the absence of the ACS (closed blue circles; *n* = 15 sessions in 4 mice). The rate of avoidance at these LCS frequencies was >70%, which is somewhat lower than ACS trials. Furthermore, a group of No Opsin mice that underwent the same procedures were not able to use the LCS effectively to avoid the US (open gray squares; *n* = 21 sessions in 4 mice). The percentage of avoidance responses during train (10–40 Hz) were significantly larger in Vgat-ChR2 mice compared with No Opsin mice (Tukey’s *t*_(52)_ > 9.2, *p* < 0.0001 [*p* < 0.0001] for each of the three frequencies).

In conclusion, increasing SNr firing to levels that do not interfere with the ability to escape the US, blocks signaled active avoidance. Conversely, in the same animal, inhibiting SNr firing drives active avoidance without any external CS. Thus, inhibiting SNr is very effective at triggering a moving away behavior.

## Discussion

In a strain of mice that express ChR2 in both GABAergic neurons and afferent fibers of the SNr, we show bidirectional control of GABAergic SNr neuron firing by adjusting the optogenetic blue light between a continuous and train pattern. In these mice, continuous blue light directly excites SNr neurons and produces ipsiversive head orienting, while trains of blue light indirectly inhibit SNr neurons by efficiently exciting GABAergic afferents which cause contraversive head orienting. Limiting ChR2 to GABAergic SNr neurons or to GABAergic afferents arriving from the striatum produced orienting head movements in only one direction regardless of the blue light pattern applied in SNr. The SNr-driven head orienting movements in opposite directions seem to have different behavioral significance because the head direction was associated with incongruent positioning of the whiskers, which only pointed in the direction of the head movement during ipsiversive orienting caused by exciting SNr neurons. Air puffs applied to the whiskers during the SNr-driven head orienting movements antagonized ipsiversive orienting but enhanced contraversive orienting, indicating that the opposite orienting head movements caused by exciting and inhibiting SNr neurons have different behavioral significance. SNr excitation seems associated with approaching or staying exploratory movements while SNr inhibition seems associated with moving away movements. Consistent with this conclusion, exciting SNr neurons suppressed a signaled goal-directed behavior that requires the animal to move away during the signal, while inhibiting SNr neurons, in the same animals, drove this behavior.

### GABAergic afferent-specific effects on orienting

We found that selective unilateral stimulation of GABAergic afferents arriving in SNr from the striatum (aka direct pathway), which inhibits SNr neurons, was very effective at producing contraversive orienting. The effect was stronger when more striatonigral fibers were stimulated because of broader striatal expression (i.e., combined dorsal and ventral striatum injections) likely reflecting afferent cooperativity in SNr. However, stimulation of GABAergic afferents from the Acb were not effective at driving orienting head movements. This is despite the fact that both of these fibers show abundant ChR2 expression in SNr and fibers from the Acb are effective at inhibiting SNr neurons (Hormigo et al., 2021). Perhaps, striatal and accumbens afferents target different populations of SNr neurons, which have different functional roles; segregation of direct pathways to SNr from different regions of striatum has been reported (Lee et al., 2020). In addition, previous work found that the GABAergic fibers originating in the Acb continue to the pedunculopontine tegmentum region (aka midbrain locomotor region) where they block signaled active avoidance when activated (Hormigo et al., 2021). Thus, GABAergic fibers reaching the midbrain from different parts of the basal ganglia likely have very distinct functional roles. It is worth noting that the low blue light power used during ACS+LCS trials in Vgat-ChR2 mice apparently were not capable of activating enough of the striatal fibers that course through SNr to the peduculopontine tegmentum. These fibers are fewer than striatal fibers terminating in SNr, and therefore require higher powers for significant activation. Increasing the light power would achieve a wider activation of these fibers but this would also drive stronger EPSPs in SNr cells in Vgat-ChR2 mice. While higher powers of blue light would recruit more of the fibers coursing through SNr to the peduculopontine tegmentum, this would also more strongly excite SNr cells during trains (i.e., ChR2-EPSPs driven by each pulse in the train), which blocks active avoidance. Thus, at the blue light powers used here we assume that we are recruiting few striatotegmental fibers that course through SNr to inhibit PPT (Hormigo et al., 2021).

### SNr and orienting significance

Animals may turn the head in the same direction to explore or to move away. The results indicate that unilateral inhibition or excitation of SNr cells not only produces orienting movements in opposite directions but those movements appear to have different behavioral significance. First, during SNr-driven head orienting, the positioning of the whiskers is not congruent with the direction of the head movement. Both ipsiversive (Cont) and contraversive (train) head movements produced protractions of the contralateral whiskers (vs the optogenetically-stimulated side). During exploration of the environment, the whiskers typically point in the direction of the head movement to evaluate objects in that direction (Towal and Hartmann, 2006). This is the case during ipsiversive head movements caused by exciting SNr, indicating that this orienting head movement may be exploratory. However, during contraversive head movements caused by inhibiting SNr, the contralateral whiskers point away from that direction in an apparent defensive reaction. Consistent with these conclusions, application of air puffs to the whiskers during head orienting movements produced distinct effects. For instance, during ipsiversive head orienting, an air puff applied on the ipsilateral side antagonized the orienting head movement indicating that this movement is exploratory; it is stopped by the annoying air puff that could signal an obstacle. In contrast, during contraversive head orienting, the air puff applied on either side enhanced the head movement, which is consistent with the idea that this is a retracting defensive movement. Contraversive orienting movements caused by inhibiting SNr appear to signify move away (retracting or defensive), while ipsiversive orienting movements caused by exciting SNr appear to signify move toward (approaching or exploratory).

The SNr on each side of the brain appears to have the capability of producing orienting movements in different directions and of different behavioral significance. Evidently, the significance and movement direction are imparted by the pattern of activation that the SNr output imposes on its target structures, such as the superior colliculus (Gandhi and Katnani, 2011), which is well known to be involved in orienting, and the thalamus (Lalive et al., 2018), which may impart significance through its projections to the neocortex. Previous work indicated that activation of direct and indirect striatal pathways (in different groups of animals), which inhibit and excite SNr neurons may be associated with reward and punishment, respectively (Kravitz et al., 2012). Those characterizations may relate to the significance imparted by SNr firing revealed in the present study. Moreover, the superior colliculus has been shown to control both approach and withdrawal orienting responses in rodents, although different terms are used to refer to these behaviors (Dean et al., 1986, 1989; Isa et al., 2020). Here, we show that the SNr already controls these types of orienting responses. Future work will address how the GABAergic SNr output controls its targets, including superior colliculus, to produce these orienting responses.

Consistent with the idea that SNr excitation and inhibition have distinct significance, we found in the same animals that exciting and inhibiting SNr can suppress or trigger a goal-directed signaled active avoidance behavior. This is consistent with our previous studies, where different groups of animals were compared (Hormigo et al., 2016, 2019, 2021). Here, we found that modulation of SNr firing (excitation or inhibition) in the same animal was able to control (suppress or facilitate) signaled active avoidance. Excitation of SNr during the CS presentation may signal the animal to stay and explore, instead of signaling to move away, which is what is required in this situation. On the other hand, inhibition of SNr during the CS presentation may facilitate active avoidance because both the ACS and the SNr inhibition (LCS) signal to move away. This raises the question of when SNr activity is excited or inhibited to produce these distinct behavioral significances. This is not known and needs investigation. One possibility is that these movements are simply a consequence of the applied stimulation and do not occur normally; typically there is a mix of SNr cells that are excited and inhibited during movement (Barter et al., 2015; Hormigo et al., 2021). Alternatively, the behavioral scenarios that would drive these different activities and significances in SNr have not been tested.

### Testing different optogenetic light patterns is useful

An important technical consideration arises from our study. Most optogenetic studies only employ one pattern of stimulation to excite circuits that express ChR2. The present findings highlight the importance of testing different light patterns to reveal possible differences in the ways circuits are impacted by these manipulations. Although, in the present study, we purposefully expressed ChR2 in different GABAergic neurons in the same circuit (e.g., SNr and striatal GABAergic neurons), intriguing differences between blue light patterns have been found even when expression is limited to specific neuronal populations (Hormigo et al., 2016, 2019, 2020, 2021). As a rule of thumb, trains of blue light may be more efficient at producing sustained effects mediated by synaptic afferents (including collaterals) that express ChR2, while continuous blue light may be more efficient at exciting somatodendritic regions that express ChR2.

## Synthesis

Reviewing Editor: Arvind Kumar, KTH Royal Institute of Technology Decisions are customarily a result of the Reviewing Editor and the peer reviewers coming together and discussing their recommendations until a consensus is reached. When revisions are invited, a fact-based synthesis statement explaining their decision and outlining what is needed to prepare a revision will be listed below. The following reviewer(s) agreed to reveal their identity: NONE.

Both the reviewers have raised substantial concerns about the manuscript at the level of presentation, methods and analysis . Following is a synthesis of specific comments from the two reviewers.

1. Integration of the work with previous research: In the last 40+ years a large body of work has been done to understand the role of SNr in shaping movements. It is reassuring that the results obtained by optical stimulation are consistent with the previous work. But we think that you have failed to integrate your work with the previous work. Crucially important work from several key researchers such as C. David Marsden, Gaetano Di Chiara, and Ian Kilpatrick to name a few, has been ignored. So, it is very important that the work is integrated with and discussed in the context of the large body of previous work. This will help clarify whether some of the results are indeed ‘remarkable’ (e.g. line 292-294) or just expected based on previous work.

2. Trial vs animal: Throughout, it is unclear what the statistical unit of analysis is. Is it trial? Is it an animal? The N for any given experiment is not always clear; and when it is known, in some cases the experimental group sizes are quite small (n=3). The statistics are reported in a manner that does not clarify this. The authors say they used ANOVA and bootstrapping, but no main effects or interaction terms are reported. So authors must explain their statistical approaches, define the N for each comparison, and completely report the stats.

3. Experimental Methods:

a. The intensity of green light is quite high. At the levels used, it seems likely that tissue was damaged. This underscores the need for histology for all experimental groups, rather than just the 7 examples from one group shown in Fig 2.

b. Compared to the behavioral experiments a weaker blue light was used for in vitro experiments. Why so? Different intensity of light stimulation may induce different responses in these neurons. Did the authors examine SNr neuronal responses induced by the same intensity of light stimulation as used in behavioral experiments?

c. Was ChR2 also expressed in GABAergic projections coming from the globus pallidus (GP)? Please clarify. And if so, discuss the possibility that light stimulation applied in the SNr excites their axon terminals too.

d. In the experiment in Figure 3B, the authors used three different intensities for light stimulation. Please mention the range of light power for “low", “medium", and “high”.

e. Page 5, line112. “The other end of the patch cables was connected to a dual light swivel that was coupled to green laser to activate Arch.” As blue light stimulation was also used to activate ChR2 and IC++ in this study, it should be mentioned with its wavelength.

4. Results:

a. In the behavioral experiment in Figure 8A, ACS+LCS stimulation at 10-40 Hz did not suppress avoidance behavior. In this study, the authors indicate that these repetitive stimuli induce GABA release from striatal axon terminals in the SNr and inhibit SNr neuronal activity. However, Hormigo et al (2021) showed that ACS+LCS stimulation applied in the SNr at 20 or 40 Hz suppressed avoidance behavior in mice in which ChR2 was expressed in the dorsal and ventral striatal neurons. What caused the difference between these studies? Please discuss it.

b. In Figure 8A, intertrial crossings and trial velocity were suppressed in all sessions with LCS. What do the results suggest? Activity of these mice decreased? Could the authors provide an explanation?

c. Authors have tested the effect of different types of activation/inactivation on orienting response in whiskers/head. Authors should discuss what these results suggest about the underlying network interactions and orientation control.

5. Text: Figures numbers in the text are not consistent and that makes reading rather difficult. Moreover, a clear narrative is missing as to why in some experiments the authors are using both excitation and inhibition approaches, transgenic and viral targeted approaches, and then in others they are using only the Vgat-ChR2. This makes the manuscript come across as a random assortment of related experiments rather than a well-linked study.

6.Figures:

a. Figure 1 is a bit cluttered. In particular panel D is very difficult to decipher. In panel F, the y-axis is rendered in Log-scale, not in semi-log. It may also help to show the continuous and pulse train stimulation in different panels so that we can see the individual trials.

b. Figure 2: Were these all of the Vgat-2 mice used in the study, or a representative subset? If the latter, authors should report the fiber optic location for each mouse. What about expression and placement for all of the other groups? A schematic for the different viral strategies would be very helpful. Confirmation of expression in striatum and accumbens, and presence of terminals in SNr should be shown.

Based on these it is important that the authors:

1. be more carefully frame their findings in the context of the large body of work done over the last 40+ years. (see Points 1,4).

2. provide a clear explanation of their statistical approaches, define the N for each comparison, and completely report the stats. (see Point 2)

3. provide missing information about the experimental methods and controls. (see Point 3)
